# Understanding the efficacy of wastewater surveillance for SARS-CoV-2 in two diverse communities

**DOI:** 10.1371/journal.pone.0289343

**Published:** 2023-08-03

**Authors:** Matthew T. Flood, Josh Sharp, Jennifer Bruggink, Molly Cormier, Bailey Gomes, Isabella Oldani, Lauren Zimmy, Joan B. Rose

**Affiliations:** 1 Department of Fisheries and Wildlife, Michigan State University, East Lansing, Michigan, United States of America; 2 Department of Biology, Northern Michigan University, Marquette, Michigan, United States of America; University of Helsinki: Helsingin Yliopisto, FINLAND

## Abstract

During the COVID-19 pandemic, wastewater-based surveillance has been shown to be a useful tool for monitoring the spread of disease in communities and the emergence of new viral variants of concern. As the pandemic enters its fourth year and clinical testing has declined, wastewater offers a consistent non-intrusive way to monitor community health in the long term. This study sought to understand how accurately wastewater monitoring represented the actual burden of disease between communities. Two communities varying in size and demographics in Michigan were monitored for SARS-CoV-2 in wastewater between March of 2020 and February of 2022. Additionally, each community was monitored for SARS-CoV-2 variants of concern from December 2020 to February 2022. Wastewater results were compared with zipcode and county level COVID-19 case data to determine which scope of clinical surveillance was most correlated with wastewater loading. Pearson *r* correlations were highest in the smaller of the two communities (population of 25,000) for N1 GC/person/day with zipcode level case data, and date of the onset of symptoms (*r* = 0.81). A clear difference was seen with more cases and virus signals in the wastewater of the larger community (population 110,000) when examined based on vaccine status, which reached only 50%. While wastewater levels of SARS-CoV-2 had a lower correlation to cases in the larger community, the information was still seen as valuable in supporting public health actions and further data including vaccination status should be examined in the future.

## Introduction

As the COVID-19 global pandemic enters its fourth year, state level surveillance of SARS-CoV- 2, the etiological agent of COVID-19 has decreased due to increased rates of self-testing and less severe disease, leading to lower reporting and underestimates of SARS-CoV-2 morbidity rates in communities. However, there remains a great interest in the use of less intrusive surveillance methods such as wastewater monitoring. A recent report by the National Academies has concluded that wastewater-based surveillance produces data that have been particularly useful for understanding the trends of the virus and the emergence of the variants associated with infection in the community [1]. A global review on the State of the Art from 26 countries found that wastewater surveillance could be used for early warning, establishing trends, estimating prevalence of infections; and studying the evolving genetics of the virus [2].

Wastewater surveillance for SARS-CoV-2 and its variants in the population provides independent and complementary data to inform public health decision-making. Because large numbers of viral particles are shed in the feces of infected individuals including symptomatic, asymptotic, and pre-symptomatic persons [3–6], molecular detection can be used to identify and quantify the viral RNA of SARS-CoV-2 in raw wastewater along with trends and spikes which have been valuable for mobilizing public health resources [1,2,7,8].

A number of studies have utilized wastewater surveillance to track the progress of COVID-19 in communities, and groups have used the sewer to focus on a single building, local area, or wastewater at the treatment facility to represent a city or county geographic scale [9–12]. Previous studies have shown that SARS-CoV-2 levels in wastewater correlate with COVID-19 cases [7,13–15]. However a study examining 5 different communities with populations ranging between 5300 to 244,100 found fairly weak correlations (spearman rank coefficients for cases by zip code versus the wastewater signals only ranged from 0.137 to 0.177) [16]. Normalization was undertaken by the pepper mild mottle virus although this did not improve the correlations, but flow and population size were not used to normalize the virus signals, In addition, variants or vaccinations were not mentioned even though sampling occurred as vaccinations in the United States began on December 14, 2020.

Understanding how differences in community size and the wastewater system impact SARS-CoV-2 wastewater results is necessary to properly apply wastewater surveillance on a wider scale. Data are now available to better understand how wastewater SARS-CoV-2 levels reflect the disease trends but the impacts of new variants and the use of vaccinations as clinical testing declines are now of great interest and data are still emerging [17,18].

The goal of this study was to determine how well wastewater surveillance for SARS-CoV-2 addressed the cases of disease in two different communities. For this purpose, two communities in Michigan were selected for comparison, one metropolitan and the other more rural. These communities vary in population size, demographics, total numbers of cases of COVID-19, and vaccination rates over the course of the early part of the pandemic. This study had four main objectives: 1) to evaluate the efficacy of wastewater monitoring of SARS-CoV-2 in two communities with diverse characteristics; 2) to compare the correlation of county cases to the zipcode level case data with wastewater surveillance results; 3) to explore the impact of vaccination rates on SARS-CoV-2 wastewater signals compared to case numbers; and 4) to examine the occurrence and appearance of new variants in sewage during the waves of COVID-19 in each community. An increased understanding of how these elements influence wastewater levels and the interpretation might give public health authorities information which could be utilized to better respond to not only SARS, but other infectious diseases in the future.

## Materials and methods

### Wastewater sampling and site descriptions

Two communities and their corresponding wastewater treatment plants were selected for sampling and comparison. A breakdown of the county level demographics, COVID-19 vaccinations, and total COVID-19 cases/1,000 persons for the two counties in this study are shown in Table 1. Population served by zipcodes and the clinical cases are the only specific data available by zipcode. Deaths for example are very sensitive and individuals could be identified especially in smaller communities, thus only county data were available. Wastewater treatment plant B (WWTP B) treats wastewater from a city and two surrounding townships within a single county. WWTP B serves a population of 25,000 persons with an average flow of 2.3 million gallons per day (MGD) (Table 1). Wastewater treatment plant A (WWTP A) serves 31 communities, with 25 within its primary county and six others communities in surrounding countiesWWTP A serves a population of 110,267 persons with an average flow of 27 MGD (Table 1). The population served has also been determined by the zip codes that the WWTP serves shown in Table 1. While most other data are only available by county/political boundaries. While both WWTP A and WWTP B use conventional activated sludge followed by disinfection however, WWTP A is an approved blending facility which handles wet weather induced inflow. This potentially increases the dilution of fecal inputs in the wastewater during wet weather events.

**Table 1:** County level demographics, COVID-19 vaccinations, and total COVID-19 cases/ 1,000 persons to date.

	County A	County B
**Total Population by County** **(Total Population by Zipcode** ^a^ **)**	405,813 (110,267)	66,699 (25,000)
**Population Density (People per sq. mile)**	637.13	36.87
**Household Size**	2.41	2.39
**Percent Living in Poverty**	19.8	16.4
**Percent of Population >65 years**	17.97	19.62
**Ratio of Male to Female Population**	48.2: 51.8	50.3: 49.7
**Ratio of White to Non-white persons**	75.3: 24.7	93.2: 6.8
**Per Capita Income (2020)**	$46,152	$44,445
**County Level GDP** **(Thousands of Current Dollars)**	16,121,115	2,787,951
**Percent Fully Vaccinated as of (5/31/22)** ^b^	50.8	64.5
**Total Number of COVID-19 Cases/1,000 persons as of 3/1/22** **(Total Number of COVID-19 Cases/1000 persons solely by zipcode as of 3/1/22)**	247 763	235 332
**Total Number of COVID-19 Deaths as of 3/1/22** **Total Number of COVID-19 Deaths/ 1,000 persons as of 3/1/22**	1,692 4.2	126 1.9

^a^Zipcodes served by WWTP;

^b^ Fully vaccinated is defined as two full doses.

Sources: [19–21].

#### Sample collection methods

Wastewater samples for this study were collected over a 24 hr period at the inflows after the primary grit removal of each WWTP. WWTP B collected composite samples based on their expected daily flow with approximately 65 ml being collected for every 58,000 gallons of wastewater entering the plant for a total of ~2500 ml for a 24 hr period. WWTP A collected composite samples based on a time paced approach collecting 100 ml every 30 mins over a 24 hr period. A total of 1 L of wastewater was then transported to the processing laboratory on ice. A total of 186 samples were collected from WWTP A (N = 92) and WWTP B (N = 94) between April 2020 and February 2022 at a frequency of once per week. Between April 2020 and January 2021, the samples from WWTP B were shipped overnight on ice to Michigan State University. Between February 2021 and December 2021, the samples from WWTP B were driven to Northern Michigan University for processing. A gap in sampling occurred for both WWTPs between January/February and May/July 2021 due to the ending of one project funding and the start of another. All samples from the WWTP A were shipped on ice overnight to Michigan State University (April 2020—December 2021). Physiological measurements including temperature, pH, biological oxygen demand (BOD), and total suspended solids (TSS) were taken at the time of sampling by each WWTP’s onsite laboratory (Table 3). Turbidity was measured upon arrival at the processing laboratory. Samples collected between April 2020 and October 2020 were kept frozen at -80°C until analysis. All samples collected after October 25^th^, 2020 were kept at 4°C, never frozen and were processed within 72 hours of collection. This change in storage temperatures was due to evidence that the SARS-CoV-2 RNA signal declined in the raw wastewater samples after they had been frozen.

### Viral concentration and processing methods

Wastewater samples were processed, and viral particles were concentrated using the polyethylene glycol (PEG) workflow published by Flood et al. (2021) [22]. Briefly, a 100 ml of sample was processed with 8% (w/vol) molecular grade PEG 8000 (Promega Corporation, Madison, Wisconsin) and 1.17 g NaCl (0.2 M w/v). After mixing, holding at 4°C, they were centrifuged at 4,700 x g at 4°C for 45 mins. Following centrifugation, the pellet was resuspended in the remaining supernatant (2–10 ml). Sample concentrates were aliquoted and either immediately underwent RNA extraction or were stored at -80°C until further processing.

Viral ribonucleic acid (RNA) was extracted using the QIAmp Viral RNA Minikit (Qiagen, Germany) according to the manufacturer’s protocol. A total of 200 μl of concentrate was used for each RNA extraction with a final elution volume of 80 μl.

### Detection and enumeration of SARS-CoV-2 from wastewater using RT-ddPCR

All genetic targets were analyzed using one-step reverse transcriptase droplet digital PCR for SARS-CoV-2 nucleocapsid 1 (N1) and nucleocapsid 2 (N2) gene targets. The primer and probe sequences for the N1, N2, and Phi6 gene targets are shown in Table 2. Samples from WWTP A were analyzed for genetic markers for SARS-CoV-2 variants of concern starting in December of 2020 using GT Molecular’s variant assay kits for digital PCR (GT Molecular, Fort Collins, Colorado, USA). These variants included the Alpha variant (gene targets N501Y and DEL69-70), the Delta variant (gene targets T478K and L452R), and the Omicron variant (gene targets N501Y, DEL69-70, and K417N). The variant assays used the same thermocycling setup as the Phi6 assay. All analyses were run with three technical replicates and a full contingent of quality controls (positive, negative, extraction negative, and non-template controls) on each assay plate. The specifics of the method are found in Flood et al [22].

**Table 2:** Primer and probe sequences.

Target	Primer/Probe name	Primer/Probe Sequence	Reference
SARS CoV-2	2019-nCoV_N1-F 2019-nCoV_N1-R 2019-nCoV_N1-P	5’-GACCCCAAAATCAGCGAAAT-3’ 5’-TCTGGTTACTGCCAGTTGAATCTG-3’ 5’-FAM-ACCCCGCATTACGTTTGGTGGACC-BHQ1-3’	[23]
	2019-nCoV_N2-F 2019-nCoV_N2-R 2019-nCoV_N2-P	5’-TTACAAACATTGGCCGCAAA-3’ 5’-GCGCGACATTCCGAAGAA-3’ 5’-HEX-ACAATTTGCCCCCAGCGCTTCAG-BHQ1-3’	[23]
Phi6	Φ6Tfor Φ6Trev Φ6Tprobe	5’-TGGCGGCGGTCAAGAGC-3’ 5’-GGATGATTCTCCAGAAGCTGCTG-3’ 5’- FAM-CGGTCGTCGCAGGTCTGACACTCGC-BHQ1-3’	[24]

### COVID-19 case and vaccination data

Data for COVID-19 cases were procured for both zipcode and county levels. Zipcode level case data were provided through an agreement with the Michigan Department of Health and Human Services (MDHHS). Zipcodes serviced by each wastewater treatment plant were provided by plant operators. Along with the case data at the zipcode level the date for onset of symptoms was provided as well as the referral date (which was the date the case was submitted to the Michigan Disease Surveillance System) for the paired data points over the course of the study. In the event of missing data for the onset of symptoms, an estimate of onset date was used based on an average of all data with known information. This was calculated by averaging the number of days between onset of symptoms and referral dates. The average number of days between onset and referral date was 5.78 days (N = 92,418) for the combined datasets (Community A + B). The average number of days between onset and referral date for each community’s zipcode level data alone were 5.93 days for A (N = 84,128) and 4.81 days for B (N = 8,290), with both ranging from 0 to 60 days.

County level case data were obtained from the US Centers for Disease Control and Prevention’s (US CDC) COVID Data Tracker website (https://data.cdc.gov/Public-Health-Surveillance/United-States-COVID-19-Community-Levels-by-County/3nnm-4jni). COVID-19 vaccination data were obtained from the US CDC’s COVID Data Tracker website (https://data.cdc.gov/Vaccinations/COVID-19-Vaccinations-in-the-United-States-County/8xkx-amqh). These data had only one date provided which was the referral date.

### Data analysis

All ddPCR results were converted from gene copies (GC) per reaction (5 μl of sample template) to GC/100 ml prior to analysis as described in Flood et al. 2021 [19,22]. Following conversion to GC/100 ml wastewater results were normalized for each community based on daily wastewater flows and zipcode level population. Non-detects (ND) replicates included in statistical analysis results were assigned their lower limits of detection for statistical analysis.

Data visualization and statistical analysis were performed using Graphpad Prism 9 (Graphpad Software, CA, USA). Correlation analyses were performed using Pearson correlation (*r*) analysis. Correlation analyses were compared for results between both the community’s wastewater results and case data, between wastewater results with zipcode specific and county level cases data, and vaccination rates and case data. To account for lag time between the wastewater signal and cases, both the date of symptom onset and the date of case referral were analyzed against the wastewater signal.

## Results

### The efficacy of SARS-CoV-2 concentrations found in wastewater against COVID-19 case data in two communities

The data gathered during this study showed that the two wastewater treatment plants (A and B) had distinctly different characteristics (Table 3). WWTP A had approximately 10 times the average daily flow (28.55 million gallons per day, MGD) of WWTP B (2.87 MGD). This is not surprising given the different populations served and the size of the system. Sample temperatures ranged from 6.5 to 22.6°C for WWTP A and samples from WWTP B ranged from 8.9 to 18.9°C. While WWTP A had slightly lower average BOD5 levels than WWTP B (168.39 and 198.69, respectively) higher turbidities were observed at WWTP A (WWTP A: 82.82 vs. WWTP B: 58.32). Wastewater N1 and N2 gene targets average concentrations for SARS-CoV-2 were similar between the two WWTPs (WWTP A (N = 106) N1 3.93, N2 3.85; WWTP B (N = 108) N1 3.93, N2 3.91 Log_10_GC/ 100ml) (Table 4). However, as expected the virus loading as calculated by daily average flow at each of the WWTPs and adjusted for population (within the zipcodes served by the WWTP) was much larger for WWTP A which had more than twice as much of the N1 gene (84.35 gene copies per person per day) compared to WWTP B (36.58 GC/Person/Day) and nearly double for the N2 gene as well (69.62 vs. 35.72 GC/Person/Day).

**Table 3:** Physiological measurements for two wastewater treatment plants.

WWTP (~miles of pipes)	Estimated Population Served by Zipcode	Flow Rate (MGD)	Temperature (°C)	pH	BOD5 (mg/L)	TSS (mg/L)	Turbidity (NTU)
A (200 to 300)	110,267	28.55 (21.10–55.68)	14.27 (6.5–22.6)	7.60 (7.28–7.97)	168.39 (60.0–500.0)	200.96 (90.0–526.0)	82.82 (25.1–158)
B (90)	25,000	2.87 (2.06–4.33)	13.76 (8.89–18.89)	7.22 (7.0–7.7)	198.69 (79.0–336.0)	194.46 (99.0–364.0)	58.32 (17.4–152.0)

Note: A gap in sampling occurred for both WWTPs between January/February and May/July 2021 due to the ending of one project funding and the start of another.

**Table 4:** Summary of wastewater monitoring results for two wastewater treatment plants.

		N1 Log_10_GC/ 100 ml	N2 Log_10_GC/ 100 ml	N1 GC/Person/ Day	N2 GC/Person/ Day
**WWTP A** **(N = 106)**	Percent Positive	74.53% (79/106)	71.70% (76/106)	74.53% (79/106)	71.70% (76/106)
	Mean^a^ (Range)	3.93 (2.70–5.07^b^)	3.85 (2.57–5.00^b^)	84.35 (3.83–1160.17)	69.62 (3.83–983.20)
**WWTP B** **(N = 108)**	Percent Positive	73.15% (79/108)	77.78% (84/108)	73.15% (67/108)	77.78% (71/108)
	Mean^a^ (Range)	3.93 (2.78–4.99^c^)	3.91 (2.78–4.95^c^)	36.58 (2.82–341.88)	35.72 (2.38–319.96)

^a^Arithmetic means;

^b^Date of peak concentration for WWTP A was 11/29/21;

^c^Date of peak concentration for WWTP B was 1/20/21;

Note: A gap in sampling occurred for both WWTPs between January/February and May/July 2021 due to the ending of one project funding and the start of another.

#### Zipcode level analysis

**Fig 1:**
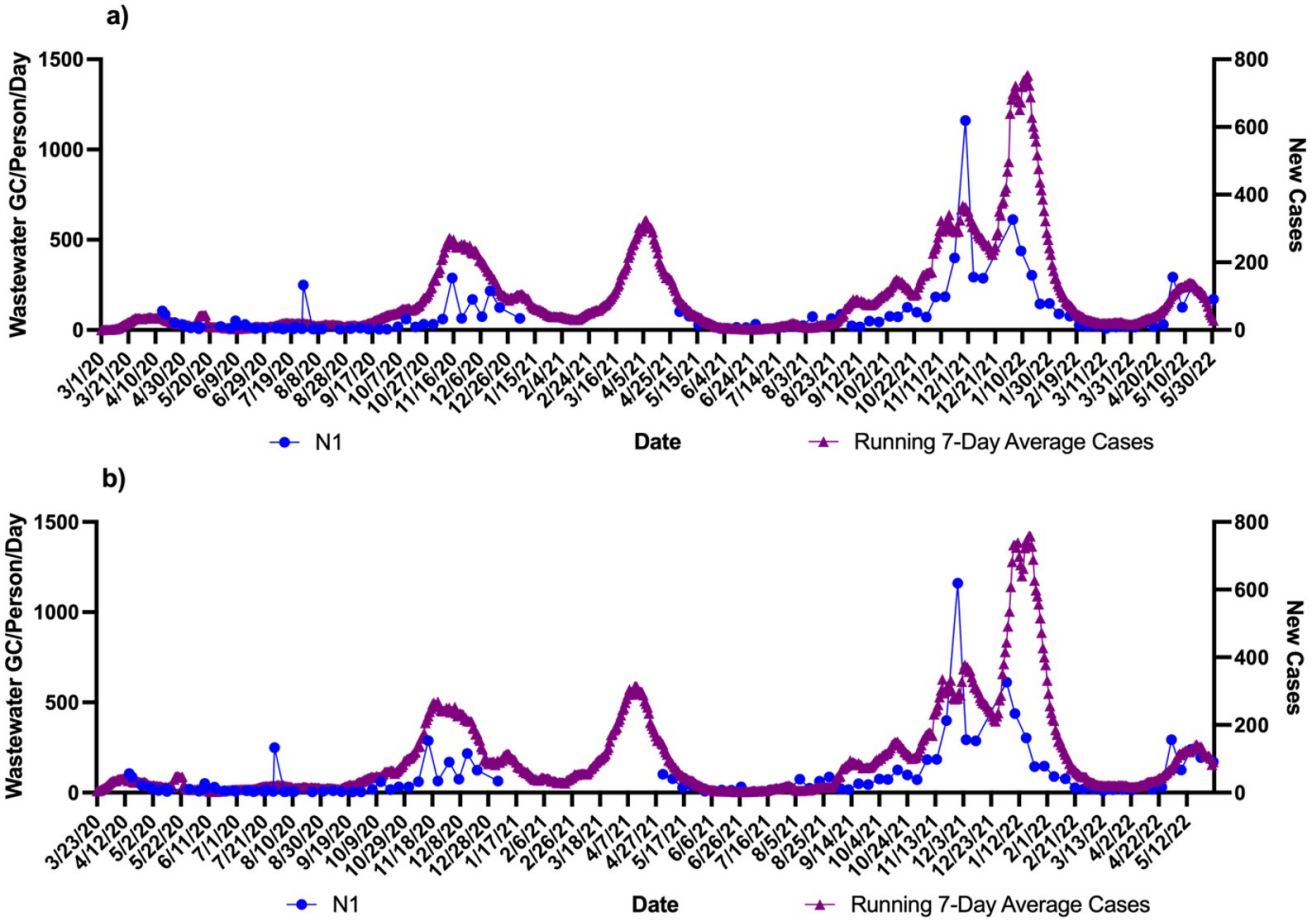
Wastewater surveillance data (N1 gene target) for WWTP A (N = 106) (GC/Person/Day) and COVID-19 zipcode case data over time. a) N1 vs. case using onset of symptoms for running 7-day average case data for COVID-19 (*r* = 0.71 p<0.0001; n = 106 paired data points); b) N1 vs. case using referral date for running 7-day average case data for COVID-19 (*r* = 0.62 p<0.0001; n = 106 paired data points). ^a^Zipcode level population data were used for wastewater results normalization; ^b^A gap in sampling occurred between January/February and May/July 2021 due to the ending of one project’s funding and the start of another.

**Fig 2:**
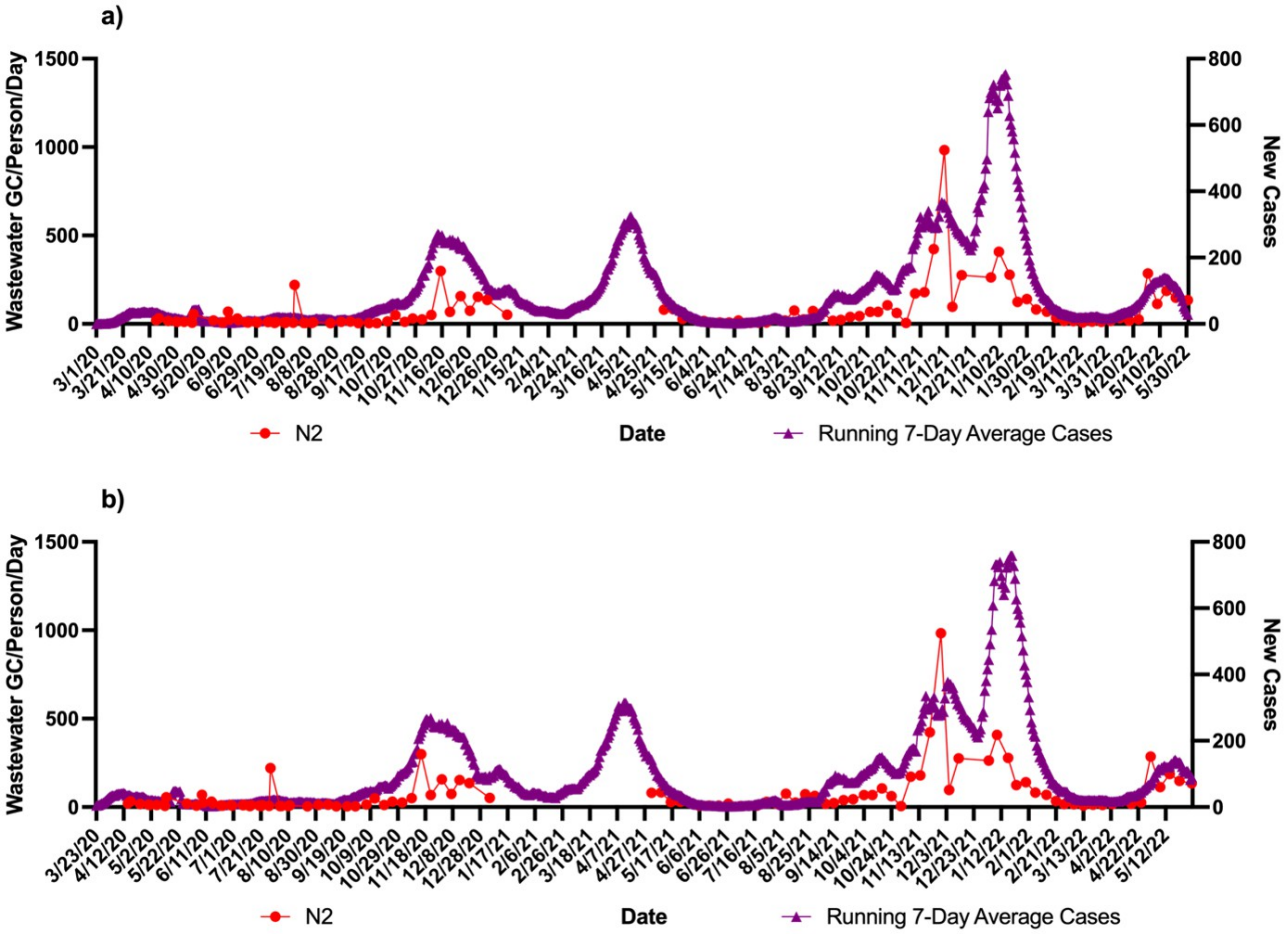
Wastewater surveillance data (N2 gene target) for WWTP A (N = 106) (GC/Person/Day) and COVID-19 zipcode case data over time. a) N2 vs. case using onset of symptoms for running 7-day average case data for COVID-19 (*r* = 0.66 p<0.0001; n = 106 paired data points); b) N2 vs. case using referral date for running 7-day average case data for COVID-19 (*r* = 0.60 p<0.0001; n = 106 paired data points). ^a^Zipcode level population data were used for wastewater results normalization; ^b^A gap in sampling occurred between January/February and May/July 2021 due to the ending of one project’s funding and the start of another.

**Fig 3:**
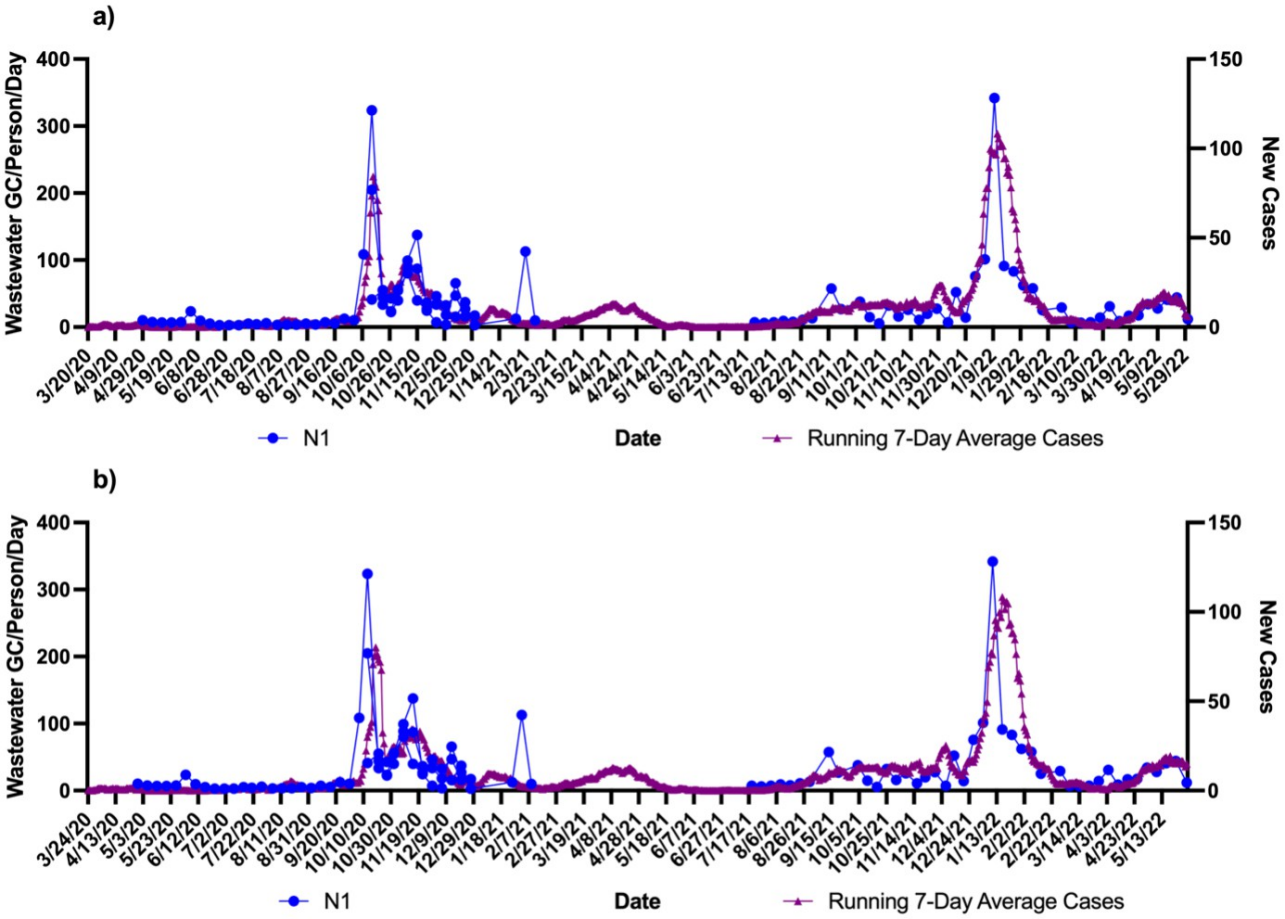
Wastewater surveillance data (N1 gene target) for WWTP B (N = 108) (GC/Person/Day) and COVID-19 zipcode case data over time. a) N1 vs. case using onset of symptoms for running 7-day average case data for COVID-19 (*r* = 0.81 p<0.0001; n = 82 paired data points); b) N1 vs. case using referral date for running 7-day average case data for COVID-19 (*r* = 0.60 p<0.0001; n = 73 paired data points). ^a^Zipcode level population data were used for wastewater results normalization; ^b^A gap in sampling occurred between January/February and May/July 2021 due to the ending of one project’s funding and the start of another.

**Fig 4:**
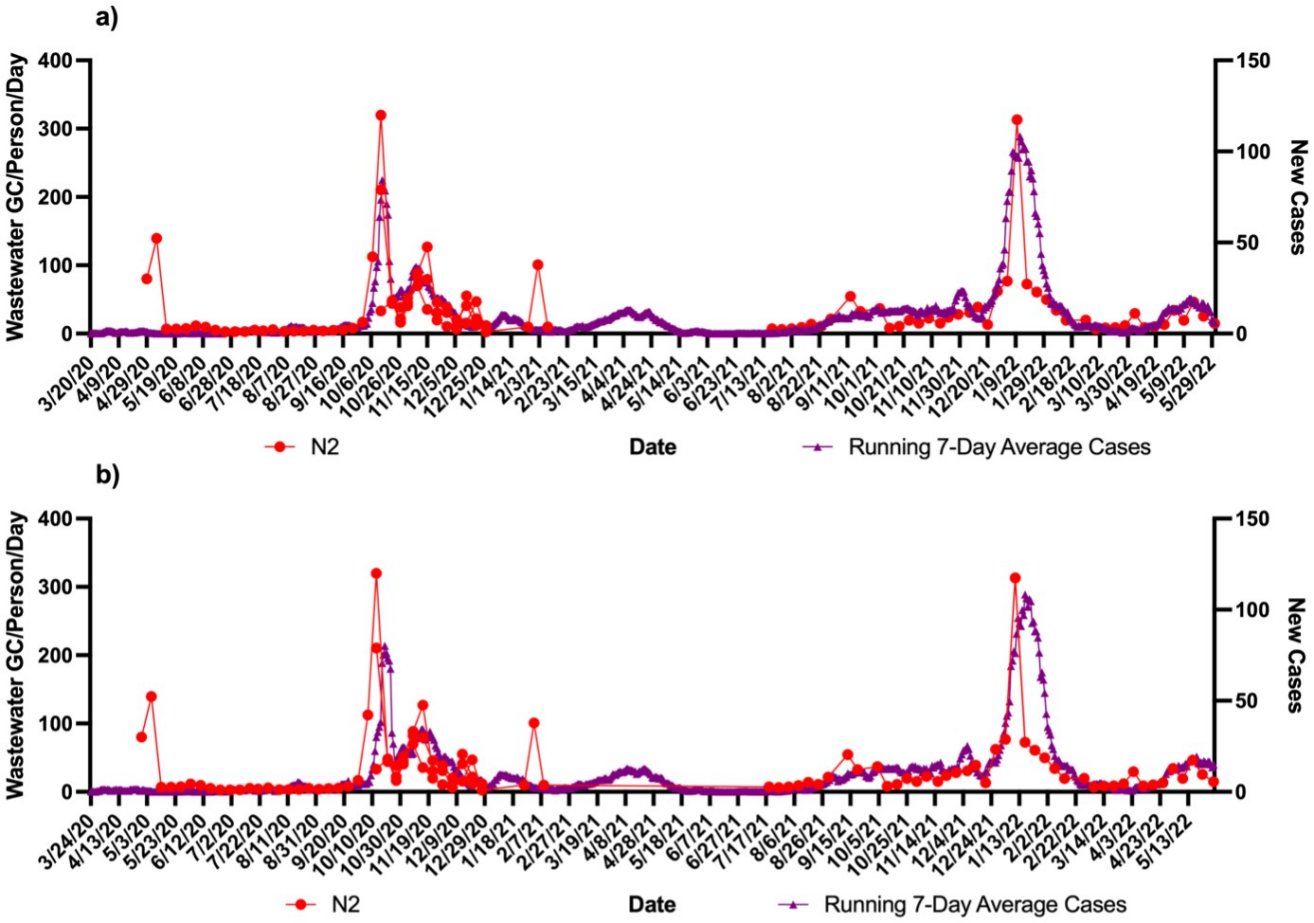
Wastewater surveillance data (N2 gene target) for WWTP B (N = 108) (GC/Person/Day) and COVID-19 zipcode case data over time. a) N2 vs. case using onset of symptoms for running 7-day average case data for COVID-19 (*r* = 0.72 p<0.0001; n = 82 paired data points); b) N2 vs. case using referral date for running 7-day average case data for COVID-19 (*r* = 0.51 p<0.0001; n = 73 paired data points). ^a^Zipcode level population data were used for wastewater results normalization; ^b^A gap in sampling occurred between January/February and May/July 2021 due to the ending of one project’s funding and the start of another.

Using the zipcodes the level of cases/1000 were 763 in community A which was twice as much as community B (332 cases/1000). These data were correlated with the wastewater signals over the course of the study. Figs 1–4 show the results of wastewater surveillance of SARS-CoV-2 graphed with the running 7-day average zipcode level case data using the the onset of symptoms date for each community compared to the date of referral for WWTP A (Figs 1 and 2 for N1; N2) and WWTP B (Figs 3 and 4 for N1; N2). A gap in wastewater data between January/February and May/July 2021 was due to the ending of one project’s funding and the start of another. The virus loading (total amount of virus per day adjusted by population) in the wastewater from both communities followed the same trends as the case data consistent with the waves of COVID-19 cases in Michigan during the pandemic. The N1 gene results for Community A had a higher correlation with the onset date (Fig 1a: *r* = 0.71 p<0.0001) compared to the referral date (Fig 1b: *r* = 0.62 p<0.0001). The N2 gene correlations for Community A were also higher for the onset compared to referral dates (Fig 2a: onset *r* = 0.66 p<0.0001; Fig 2b: referral *r* = 0.60 p<0.0001).

A larger difference in correlations was observed with Community B (Figs 3 and 4). The N1 gene results had the highest correlation observed in the study with the onset date (Fig 3a: *r* = 0.81 p<0.0001) compared to the referral date (Fig 3b: *r* = 0.60 p<0.0001). This same pattern was seen with the N2 gene results as well with the onset date (Fig 4a), showing a correlation of *r* = 0.72 (p<0.0001) while the referral date (Fig 4b) was only *r* = 0.51 (p<0.0001).

### Zipcode vs county level case data varying spatial resolution

**Fig 5:**
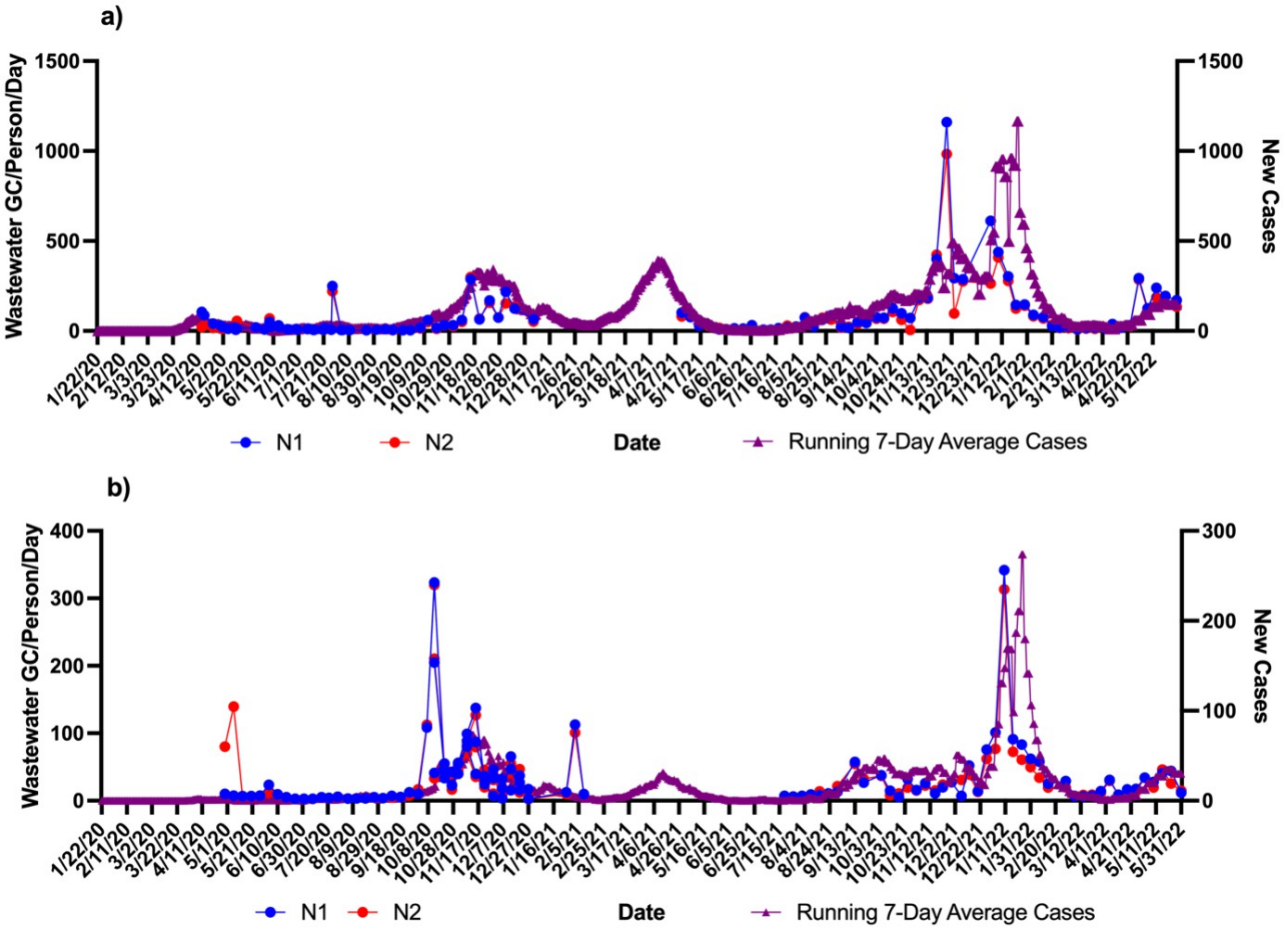
Wastewater surveillance data (N = 108) (GC/person/day using zipcode level population) compared to county level COVID-19 clinical case data over time using referral dates only. a) WWTP A SARS-CoV-2 gene target results vs. county level case data for COVID-19 (N1 *r* = 0.59 p<0.0001, N2 *r* = 0.56 p<0.0001; n = 106 paired data points); b) WWTP B SARS-CoV-2 gene target results vs. county level COVID-19 case data (N1 *r* = 0.53 p<0.0001, N2 *r* = 0.46 p<0.0001; n = 73 paired data points). ^a^Zipcode level population data were used for wastewater results normalization; ^b^A gap in sampling occurred between January/February and May/July 2021 due to the ending of one project funding and the start of another.

The county level clinical case data had only one date associated with the cases (referral date), thus the correlations for two communities evaluated county level case data (referral dates) against the virus levels. The two communities showed similar pearson correlation values of approximately 0.5 (WWTP A: N1 *r* = 0.59 p<0.0001, N2 *r* = 0.56 p<0.0001; n = 106 paired data points; WWTP B N1 *r* = 0.53 p<0.0001, N2 *r* = 0.46 p<0.0001; n = 73 paired data points) (Fig 5). Although like the previous analysis the N2 gene showed a lower correlation compared to N1. It is important to note that the discrepancies in the total paired data points and the paired data points in the county level data were due the presence of censored data for multiple dates in the US CDC database.

The zipcode level case data represented 25% of county level case data for Community A and 37.5% for Community B. At the county level more cases were reported in the clinical database whose sewage was not associated with the WWTP. Thus, it is not surprising that the correlations were higher using zipcode case level case data (even when using the referral dates), compared to using County level case data for Community A, where N1 showed a correlation of *r* = 0.62 (p<0.0001) compared to *r* = 0.59 (p<0.0001). But for Community B, the correlations were the same.

Regardless of the size of the community the N1 gene levels (using GC/person/day), use of zipcode case level data representing the WWTP and the date of onset of illness improve an understanding of the relationship between virus levels in wastewater and cases of disease in the community.

### Impact of vaccination rates on SARS-CoV-2 wastewater signals and case numbers

**Fig 6:**
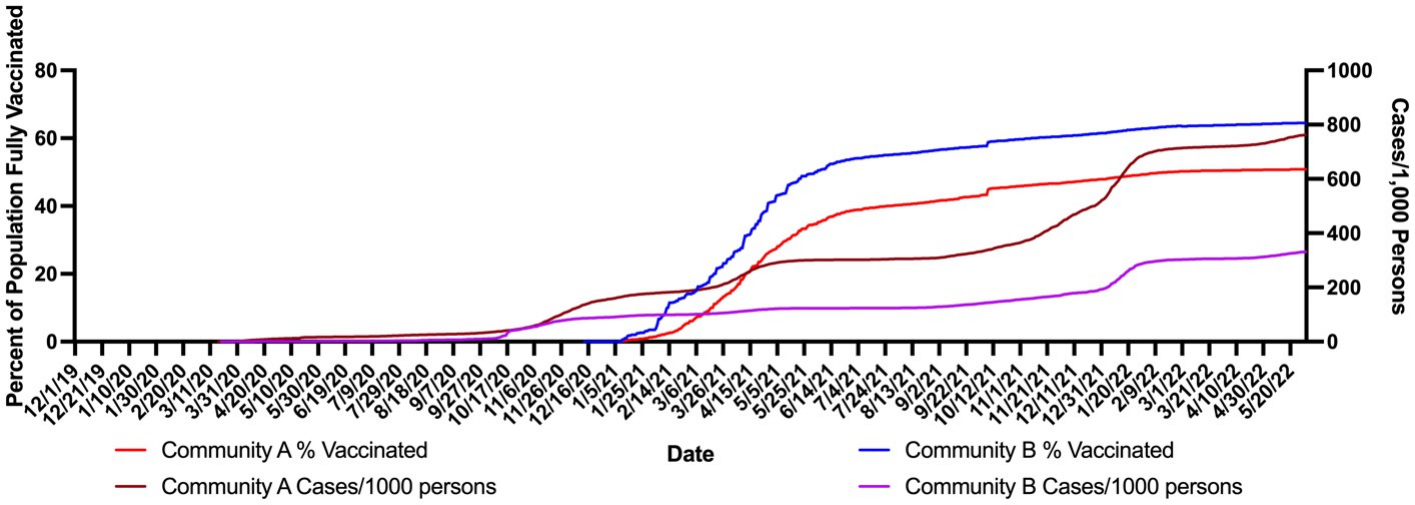
Percent of population fully vaccinated compared with county level cases per 1,000 persons for Community A and Community B.

**Fig 7:**
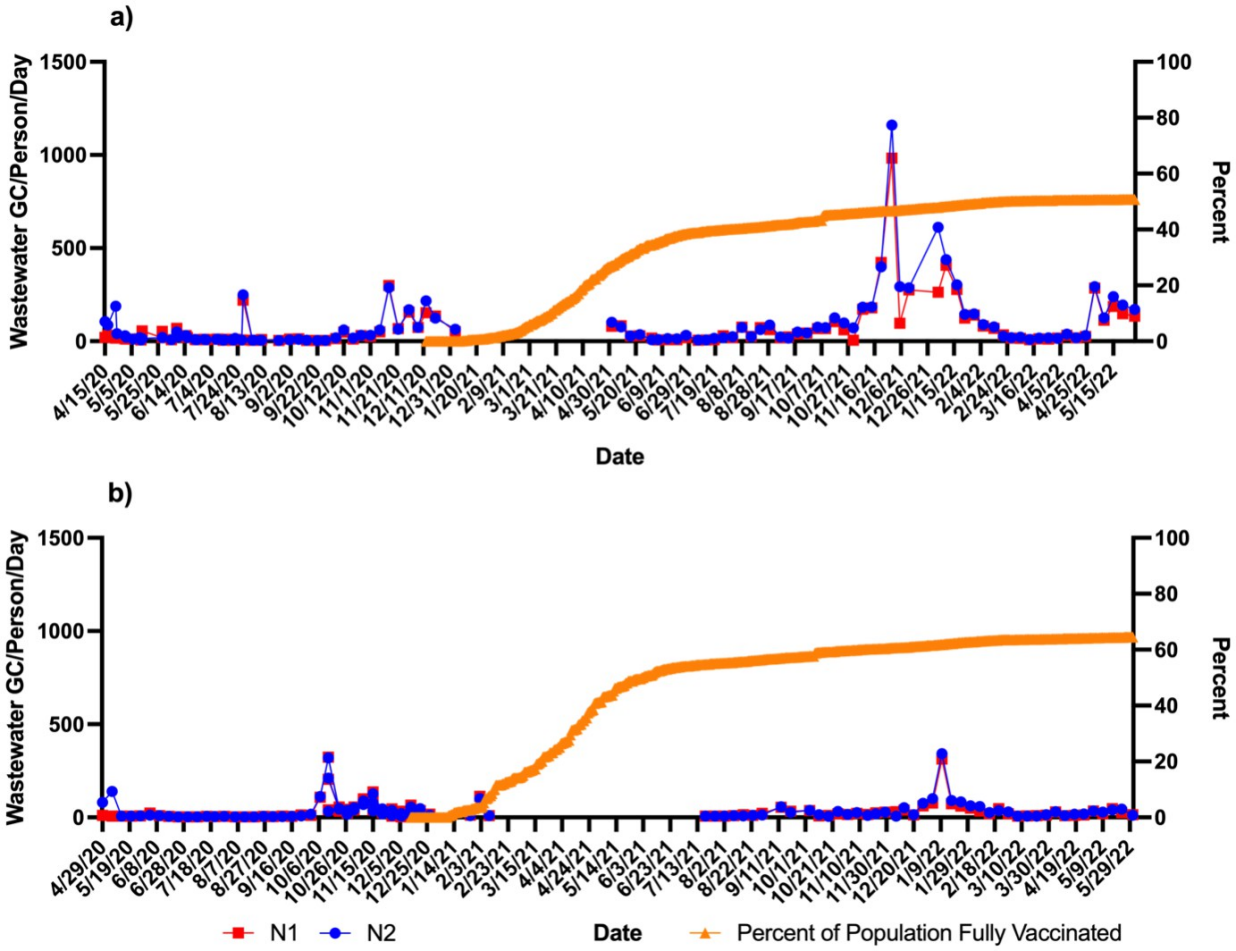
Percent of population fully vaccinated compared with wastewater SARS-CoV-2 gene targets (N1 and N2 (GC/Person/Day). a) WWTP A; b) WWTP B. ^a^Zipcode level population data were used for wastewater results normalization; ^b^A gap in sampling occurred between January/February and May/July 2021 due to the ending of one project’s funding and the start of another.

In this study, the percent of the population vaccinated at the county level were graphed per day. Vaccination data in this case were for that proportion of the population fully vaccinated (defined as two doses) for the two counties served by WWTP A and B. The first reported data point for vaccination rate was in December of 2020. Both counties had rapid increases in vaccination over the following six months with Community A reaching 34.7% and Community B reaching 50% of persons fully vaccinated by June 2, 2021 (Fig 6). However, after June of 2021 the vaccination rates slowed and began to plateau. While it took 172 days for Community B to reach 50% vaccination, Community A took 2.5 times as long reaching 50% of its populations fully vaccinated at 433 days. The final vaccination rate for each community at the end of the study period (May 31, 2022) was 50.8% for Community A and 64.5% for Community B. While Community B had lower cases/ 1000 persons than Community A vaccinations began in Community B almost two months before Community A (Fig 6). The cases in Community A cases/ 1000 persons continued to rise at a steeper rate than in Community B (Fig 6). Fig 7 shows the wastewater levels (GC/person/day) and percent of the population does not show a statistical inverse relationship in either community, however as the percentage of the population vaccinated plateaus there are obvious higher spikes in the wastewater levels in Community A compared to Community B. Thus in Community A reaching only a maximum of about 50% vaccination experienced more cases and had an increase in the wastewater signal between November (2021) and March (2022).

### Detection of SARS-CoV-2 variants in wastewater over time

**Fig 8:**
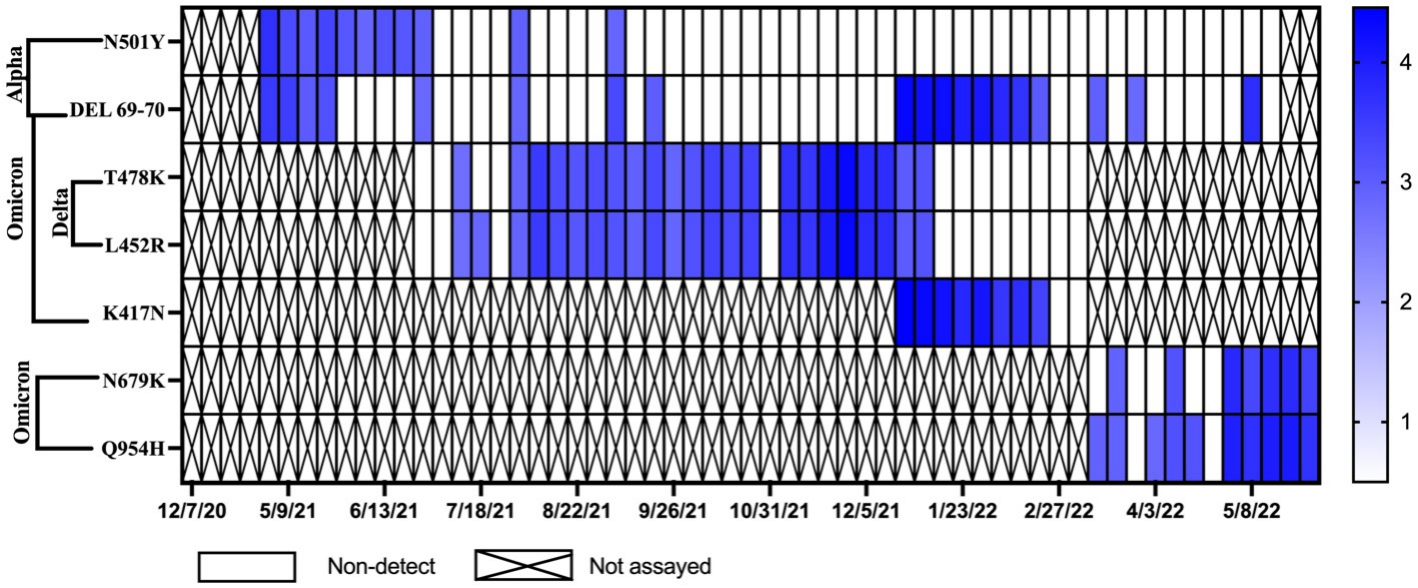
Concentrations of SARS-CoV-2 variant genes for the Alpha, Delta, and Omicron variants over time in Community A. Samples positive for the N501Y and DEL 69–70 gene mutations indicate the potential presence of the Alpha variant. Samples positive for the T478K and L452R gene mutations indicate the presence of the Delta variant. Samples positive for the K417N and DEL 69–70 gene mutations indicate the presence of the Omicron variant. Empty squares represent Non-detects (NDs) and X’s were samples that were not assayed for that marker.

**Fig 9:**
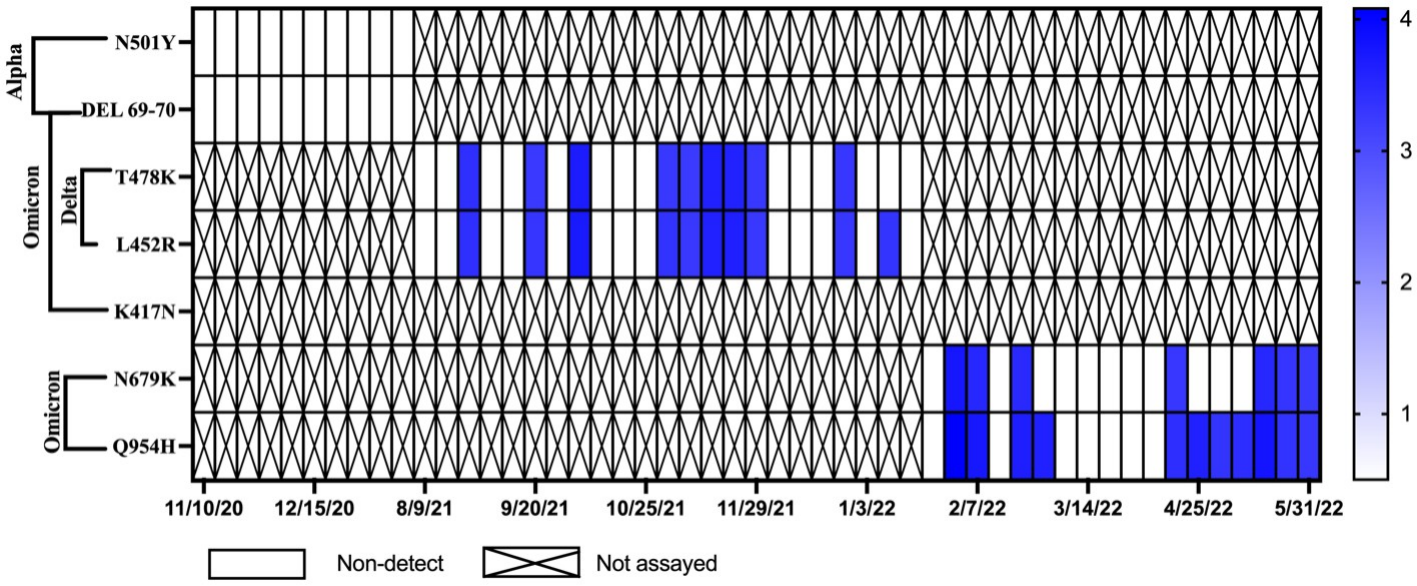
Concentrations of SARS-CoV-2 variant genes for the Alpha, Delta, and Omicron variants over time in Community B. Samples positive for the N501Y and DEL 69–70 gene mutations indicate the potential presence of the Alpha variant. Samples positive for the T478K and L452R gene mutations indicate the presence of the Delta variant. Samples positive for the K417N and DEL 69–70 gene mutations indicate the presence of the Omicron variant. Empty squares represent Non-detects (NDs) and X’s were samples that were not assayed for that marker.

Monitoring for SARS-CoV-2 variants of concern for WWTP A and B began in December of 2020 with initial testing for the Alpha variant. In June of 2021, samples from WWTP A began to be monitored for mutations associated with the Delta variant and subsequently in January of 2022 samples were monitored for mutations associated with the Omicron variant (Fig 8). The N501Y and DEL 69–70 mutations, which indicate the potential presence of the Alpha variant, were first detected in WWTP A in May of 2021. During this same period the Alpha variant mutations were not detected in samples from WWTP B (Fig 9). Levels of N501Y and DEL 69–70 in WWTP A declined as the Delta variant began to spread in Michigan in June of 2021. In the state of Michigan, the Delta variant was first detected in clinical samples and confirmed by genetic sequencing on January 16, 2021. However, it was not until July 12, 2021, that the Delta variant genes (T478K and L452R) were detected in samples from WWTP A (Fig 8). Delta variant genes were detected in WWTP B a month later on August 23, 2021 (Fig 9). Detection of Alpha variant genes declined in WWTP A as the Delta variant spread. Delta variant mutations remained dominant in wastewater samples from both communities until January 9, 2022. The K417N and DEL 69–70 mutations, indicative of the Omicron variant, were first detected in the state of Michigan on December 1, 2021. The Omicron variant was detected in WWTP A on January 3, 2022. In January of 2022, analyses for the Omicron variant were transitioned to the N679K and Q954H gene targets. These two targets were first detected in WWTP B on January 31, 2022.

## Discussion

This study demonstrated that, regardless of the size of the community, targeting N1 gene levels (using GC/person/day), using zipcode case level data representing the WWTP and the date of onset of symptoms improved the relationship between virus levels in wastewater and cases of disease in the community. Community B with the smaller population had the best correlation (r = 0.81). Besides the size of the population a further complication which may have led to lower correlations was due to septage that was brought into WWTP A. According to the utilities no septage was accepted at WWTP B but 3,265 trucks were received in 2022 for WWTP A for a total of 10.6 million gallons. While there is currently only one study that has investigated SARS-CoV-2 in septic tanks [25] it was focused on treatment and disinfection of hospital wastewater. There is currently no information on the stability of the signal in septage as this would greatly influence what might be expected in individual household wastewater in septic tanks, pumped and brought to the WWTP.

While, the communities had similar household size and per capita income, county level GDP was higher in Community A, despite the rate of poverty in Community A being slightly higher (19.8% vs. 16.4% for Community B). Community A had a higher ratio of non-white to white persons. Thus the population in Community A appeared to be more susceptible to the spread of COVID19. While at the county level it looked like the cases of disease were similar, at the zipcode level the total number of COVID-19 Cases/1,000 persons as of 3/1/22 was 763 in Community A compared to the 332 in Community B. This was more commensurate with the mortality (4.2 versus 1.4 deaths/1000 persons, respectively for Community A and B). Yet according to the State Health Department testing was 1.5 times higher in Community B compared to Community A, so this could have influenced the cases identified and improved the numbers reported in Community B.

Additionally, as of May 31^st^ 2022 only 50.8% of Community A were fully vaccinated versus 64.5% of Community B. This appeared to influence the cases and wastewater signals. While earlier vaccination rate increases, and lower population levels may have helped curb the increase in cases in Community B compared to Community A, but the current data set was insufficient to statistically evaluate the change in the rate of new cases and increasing virus levels in the wastewater. This was also complicated by the emergence of new variants. In Community A the gene targets for the Alpha variant were present consistently from May 2, 2021 until June 27, 2021 over which time the vaccination rate for the community increased from 29.3 to 38.4%. After June 27^th^, the Alpha variant genes were mostly absent from the wastewater samples and were replaced by the Delta variant mutations. These results are similar to those seen by Yaniv et al. [26], where an increase in vaccination rates was correlated with the decrease in the prevalence of the Alpha variant, but not the more infectious Delta variant.

Measuring case severity, such as hospitalizations or mortality rates, may be a better marker to evaluate the impact of vaccination in communities. Yet due to the sensitivity of the data the deaths were censored and could not be analyzed. Strict human subjects agreements would need to developed to further examine the deaths against wastewater signals. The inability to distinguish whether the disease, hospital or death cases were associated with the vaccinated or unvaccinated individuals was also a limiting factor in accurate examination of the results [27]. Community A had a much greater mortality than Community B over the course of this study. This may have been influenced by access to health care in the greater minority community and as represented by the lower vaccination rates [28]. Hospitalization data were not available at the time of the study for download by county or zipcode temporally from the US CDC or state COVID-19 database. The lag between deaths and diagnosis is highly variable, a mean of 18.1 days was reported where the estimated 90% percentile of time to death was 33.3 days [29]. This information could be requested for future analysis. It is clear that increases in COVID-19 cases are represented by increases in SARS-CoV-2 GC/person/day in sewage and this should be considered a warning signal for these disadvantaged communities with lower vaccination rates and should mobilize health care resources for variants eliciting greater severity.

Various methods have been used to statistically relate cases of COVID-19 to SARS-CoV-2 concentrations in sewage. Feng et al. [30] and Ai et al. [31] have found that use of a fecal indicator does not necessarily improve the correlations. However, Mazumder et al. [32] and Feng et al. [30] have found that normalization using loading of the virus per day by population improved the comparisons, which was used in this study. The lag between when cases were reported and when the onset of symptoms occurred was examined by using the onset of symptoms compared to the date of case referral. Larger complex communities are more difficult to monitor, and detection limits of the cases/1000 persons associated with the wastewater signal need to be further investigated.

Wastewater monitoring for the virus, the surveillance of variant gene targets provided valuable information about waves of new COVID-19 cases in the communities. While the size of each community did not affect the ability of new variants to spread to them, each new wave of variant (alpha, delta, and omicron) was first seen in Community A. While this may be due to the population size and density of Community A compared to B, the physical locations of each may have had a more significant impact. Community B was more physically isolated from other communities compared to Community A.

Wastewater monitoring during the COVID-19 pandemic has successfully demonstrated its ability for non-intrusive community health disease surveillance. In the state of Michigan this provided valuable information for public health authorities to utilize alongside clinical testing. This value has also been recognized by the US CDC, which has moved forward with efforts to collect and centralize wastewater monitoring data from across the country. The use for wastewater monitoring in the future is not limited to COVID-19. The results of this study suggest that wastewater surveillance to be more representative of cases should be presented at the higher spatial resolution of cases at the zipcode level and could then be better tied to the onset of symptoms. Using this approach to be able to routinely monitor entire communities for other infectious diseases may allow public health authorities to collect information which could help them better respond to public health crises in the future.

## Supporting information

S1 File (XLSX)

## References

[1] National Academies of Sciences, Engineering, and Medicine. 2023. Wastewater-based Disease Surveillance for Public Health Action. Washington, DC: The National Academies Press. 37184191

[2] Ferraro GB; Veneri C; Mancini P; Iaconelli M; Suffredini E; Bonadonna L; et al. A State-of-the-Art Scoping Review on SARS-CoV-2 in Sewage Focusing on the Potential of Wastewater Surveillance for the Monitoring of the COVID-19 Pandemic 2022 Food and Environmental Virology Volume14 Issue4, Page 315–354. doi: 10.1007/s12560-021-09498-6 34727334 PMC8561373

[3] Parasa S., Desai M., Chandrasekar V.T., Patel H.K., Kennedy K.F., Roesch T., et al. Prevalence of Gastrointestinal Symptoms and Fecal Viral Shedding in Patients with Coronavirus Disease 2019. Jama Netw Open 2020;3: e2011335. doi: 10.1001/jamanetworkopen.2020.11335 32525549 PMC7290409

[4] Wang W., Xu Y., Gao R., Lu R., Han K., Wu G., et al. Detection of SARS-CoV-2 in Different Types of Clinical Specimens. Jama 2020;323: 1843–1844. doi: 10.1001/jama.2020.3786 32159775 PMC7066521

[5] Zheng S., Fan J., Yu F., Feng B., Lou B., Zou Q., et al. Viral Load Dynamics and Disease Severity in Patients Infected with SARS-CoV-2 in Zhejiang Province, China, January-March 2020: Retrospective Cohort Study. Bmj 2020;369: m1443. doi: 10.1136/bmj.m1443 32317267 PMC7190077

[6] Lee S., Kim T., Lee E., Lee C., Kim H., Rhee H., et al. Clinical Course and Molecular Viral Shedding Among Asymptomatic and Symptomatic Patients With SARS-CoV-2 Infection in a Community Treatment Center in the Republic of Korea. Jama Intern Med 2020;180: 1447–1452. doi: 10.1001/jamainternmed.2020.3862 32780793 PMC7411944

[7] Peccia J., Zulli A., Brackney D.E., Grubaugh N.D., Kaplan E.H., Casanovas-Massana A., et al. Measurement of SARS-CoV-2 RNA in Wastewater Tracks Community Infection Dynamics. Nat Biotechnol 2020;38: 1164–1167. doi: 10.1038/s41587-020-0684-z 32948856 PMC8325066

[8] Medema G., Heijnen L., Elsinga G., Italiaander R., and Brouwer A. Presence of SARS-Coronavirus-2 RNA in Sewage and Correlation with Reported COVID-19 Prevalence in the Early Stage of the Epidemic in The Netherlands. Environ Sci Tech Let 2020;7: 511–516. doi: 10.1021/acs.estlett.0c00357 37566285

[9] Fahrenfeld N.L., Medina W.R.M., D’Elia S., Modica M., Ruiz A., and McLane M. Comparison of Residential Dormitory COVID-19 Monitoring via Weekly Saliva Testing and Sewage Monitoring. Sci Total Environ 2022;814: 151947–151947. doi: 10.1016/j.scitotenv.2021.151947 34838560 PMC8611854

[10] Rasero F.J.R., Ruano L.A.M., Real P.R.D., Gómez L.C., and Lorusso N. Associations Between SARS-CoV-2 RNA Concentrations in Wastewater and COVID-19 Rates in Days After Sampling in Small Urban Areas of Seville: A Time Series Study. Sci Total Environ 2022;806: 150573. doi: 10.1016/j.scitotenv.2021.150573 34582878 PMC8464400

[11] Lastra A., Botello J., Pinilla A., Urrutia J.I., Canora J., Sánchez J., et al. SARS-CoV-2 Detection in Wastewater as an Early Warning Indicator for COVID-19 Pandemic. Madrid Region Case Study. Environ Res 2022;203: 111852–111852. doi: 10.1016/j.envres.2021.111852 34364862 PMC8342901

[12] Layton B.A., Kaya D., Kelly C., Williamson K.J., Alegre D., Bachhuber S.M., et al. Evaluation of a Wastewater-Based Epidemiological Approach to Estimate the Prevalence of SARS-CoV-2 Infections and the Detection of Viral Variants in Disparate Oregon Communities at City and Neighborhood Scales. Environ Health Persp 2022;130: 067010. doi: 10.1289/EHP10289 35767012 PMC9241984

[13] Gonzalez R., Curtis K., Bivins A., Bibby K., Weir M.H., Yetka K., et al. COVID-19 Surveillance in Southeastern Virginia using Wastewater-based Epidemiology. Water Res 2020;186: 116296. doi: 10.1016/j.watres.2020.116296 32841929 PMC7424388

[14] Gerrity D., Papp K., Stoker M., Sims A., and Frehner W. Early-pandemic Wastewater Surveillance of SARS-CoV-2 in Southern Nevada: Methodology, Occurrence, and Incidence/Prevalence Considerations. Water Res X 2021;10: 100086. doi: 10.1016/j.wroa.2020.100086 33398255 PMC7774458

[15] Graham K.E., Loeb S.K., Wolfe M.K., Catoe D., Sinnott-Armstrong N., Kim S., et al. SARS-CoV–2 RNA in Wastewater Settled Solids Is Associated with COVID-19 Cases in a Large Urban Sewershed. Environ Sci Technol 2021;55: 488–498. doi: 10.1021/acs.est.0c06191 33283515

[16] Wartell BA; Ballare S; Ghandehari SS; Arcellana PD; Proano C; Kaya D.; et al. Relationship between SARS-CoV-2 in wastewater and clinical data from five wastewater sheds. Journal of Hazardous Materials Advances, 2022 Volume 8, Page100159 doi: 10.1016/j.hazadv.2022.100159 36619827 PMC9448702

[17] Martin J., Klapsa D., Wilton T., Zambon M., Bentley E., Bujaki E., et al. Tracking SARS-CoV-2 in Sewage: Evidence of Changes in Virus Variant Predominance during COVID-19 Pandemic. Viruses 2020;12: 1144. doi: 10.3390/v12101144 33050264 PMC7601348

[18] Smith T., Cassell G., and Bhatnagar A. Wastewater Surveillance Can Have a Second Act in COVID-19 Vaccine Distribution. Jama Heal Forum 2021;2: e201616. doi: 10.1001/jamahealthforum.2020.1616 36218425

[19] US Centers for Disease Control and Prevention (CDC). COVID Data Tracker. US Centers for Disease Control and Prevention. 2022. https://covid.cdc.gov/covid-data-tracker/#datatracker-home.

[20] Bureau of Econmic Affairs (BEA). Personal Income by County, Metro, and Other Areas. 2022. https://www.bea.gov/data/income-saving/personal-income-county-metro-and-other-areas.

[21] US Census Bureau. County Population by Characteristics: 2010–2019. United States Census Bureau Website. 2022. https://www.census.gov/data/tables/time-series/demo/popest/2010s-counties-detail.html.

[22] Flood M.T., D’Souza N., Rose J.B., and Aw T.G. Methods Evaluation for Rapid Concentration and Quantification of SARS-CoV-2 in Raw Wastewater Using Droplet Digital and Quantitative RT-PCR. Food and environmental virology. 2021;13(3): 303–315. doi: 10.1007/s12560-021-09488-8 34296387 PMC8297606

[23] US Centers for Disease Control and Prevention (CDC). 2019-Novel Coronavirus (2019-nCoV) Real-time rRT-PCR Panel. 2020.

[24] Gendron L., Verreault D., Veillette M., Moineau S., and Duchaine C. Evaluation of Filters for the Sampling and Quantification of RNA Phage Aerosols. Aerosol Sci Tech 2010;44: 893–901. doi: 10.1080/02786826.2010.501351

[25] Zhang D., Ling H., Huang X., Li J., Li W., Yi C., et al. Potential Spreading Risks and Disinfection Challenges of Medical Wastewater by the Presence of Severe Acute Respiratory Syndrome Coronavirus 2 (SARS-CoV-2) Viral RNA in Septic Tanks of Fangcang Hospital. Sci Total Environ 2020;741: 140445. doi: 10.1016/j.scitotenv.2020.140445 32599407 PMC7308756

[26] Yaniv K., Ozer E., Lewis Y., and Kushmaro A. RT-qPCR Sssays for SARS-CoV-2 Variants of Concern in Wastewater Reveals Compromised Vaccination-induced Immunity. Water Res 2021;207: 117808–117808. doi: 10.1016/j.watres.2021.117808 34753092 PMC8551083

[27] Rainey A.L., Loeb J.C., Robinson S.E., Lednicky J.A., McPherson J., Colson S., et al. Wastewater Surveillance for SARS-CoV-2 in a Small Coastal Community: Effects of Tourism on Viral Presence and Variant Identification Among Low Prevalence Populations. Environ Res 2022;208: 112496–112496. doi: 10.1016/j.envres.2021.112496 34902379 PMC8820684

[28] Alcendor D.J. Racial Disparities-Associated COVID-19 Mortality among Minority Populations in the US. J Clin Medicine 2020;9: 2442. doi: 10.3390/jcm9082442 32751633 PMC7466083

[29] Marschner I.C. Estimating age‑specific COVID‑19 fatality risk and time to death by comparing population diagnosis and death patterns: Australian data. BMC Med Res Methodol. 2021; 21:126 doi: 10.1186/s12874-021-01314-w 34154563 PMC8215490

[30] Feng S., Roguet A., McClary-Gutierrez J.S., Newton R.J., Kloczko N., Meiman J.G., et al. Evaluation of Sampling, Analysis, and Normalization Methods for SARS-CoV–2 Concentrations in Wastewater to Assess COVID-19 Burdens in Wisconsin Communities. Acs Es T Water 2021;1: 1955–1965. doi: 10.1021/acsestwater.1c00160

[31] Ai Y., Davis A., Jones D., Lemeshow S., Tu H., He F., et al. (2021). Wastewater SARS-CoV-2 Monitoring as a Community-level COVID-19 Trend Tracker and Variants in Ohio, United States. Sci Total Environ 2021;801: 149757. doi: 10.1016/j.scitotenv.2021.149757 34467932 PMC8373851

[32] Mazumder P., Dash S., Honda R., Sonne C., and Kumar M. Sewage surveillance for SARS-CoV-2: Molecular Detection, Quantification and Normalization Factors. Curr Opin Environ Sci Heal 2022;100363. doi: 10.1016/j.coesh.2022.100363 35694049 PMC9170178

